# Excessive fibroblast growth factor 23 promotes renal fibrosis in mice with type 2 cardiorenal syndrome

**DOI:** 10.18632/aging.202448

**Published:** 2021-01-15

**Authors:** Huixin Hao, Siyuan Ma, Cankun Zheng, Qiancheng Wang, Hairuo Lin, Zhenhuan Chen, Jiahe Xie, Lin Chen, Kaitong Chen, Yuegang Wang, Xiaobo Huang, Shiping Cao, Wangjun Liao, Jianping Bin, Yulin Liao

**Affiliations:** 1 Department of Cardiology, State Key Laboratory of Organ Failure Research, Guangdong Provincial Key Lab of Shock and Microcirculation, Nanfang Hospital, Southern Medical University, Guangzhou 510515, China; 2 Department of Oncology, Nanfang Hospital, Southern Medical University, Guangzhou 510515, China

**Keywords:** fibroblast growth factor 23, myocardial infarction, cardiorenal dysfunction, fibrosis

## Abstract

Cardiorenal syndrome (CRS) has a high mortality, but its pathogenesis remains elusive. Fibroblast growth factor 23 (FGF23) is increased in both renal dysfunction and cardiac dysfunction, and FGF receptor 4 (FGFR4) has been identified as a receptor for FGF23. Deficiency of FGF23 causes growth retardation and shortens the lifespan, but it is unclear whether excess FGF23 is detrimental in CRS. This study sought to investigate whether FGF23 plays an important role in CRS-induced renal fibrosis. A mouse model of CRS was created by surgical myocardial infarction for 12 weeks. CRS mice showed a significant increase of circulatory and renal FGF23 protein levels, as well as an upregulation of p-GSK, active-β-catenin, TGF-β, collagen I and vimentin, a downregulation of renal Klotho expression and induction of cardiorenal dysfunction and cardiorenal fibrosis. These changes were enhanced by cardiac overexpression of FGF23 and attenuated by FGF receptor blocker PD173074 or β-catenin blocker IGC001. In fibroblasts (NRK-49F), expression of FGFR4 rather than Klotho was detected. Recombinant FGF23 upregulated the expression of p-GSK, active-β-catenin, TGF-β, collagen I and vimentin proteins. These changes were attenuated by FGFR4 blockade with BLU9931 or β-catenin blockade with IGC001. We concluded that FGF23 promotes CRS-induced renal fibrosis mediated by partly activating FGFR4/β-catenin signaling pathway.

## INTRODUCTION

Cardiorenal syndrome (CRS) is a pathophysiologic disorder of the heart and kidneys, whereby acute or chronic dysfunction in one organ induces acute or chronic dysfunction in the other organ. In 2008, Ronco et al. proposed five subtypes of CRS according to the temporal sequence of organ failure as well as the clinical context [1]. CRS type 1 or acute cardiorenal syndrome (CRS-1) is characterized by an acute cardiac disease leading to acute kidney injury. The most common etiologies for an acute cardiac disease include acute decompensated heart failure, acute coronary syndrome and cardiac surgery [2]. Myocardial infarction (MI) may lead to cardiorenal dysfunction, which is termed CRS and is recognized as a major public health problem due to its high morbidity and mortality [3, 4]. Type 2 CRS is characterized by chronic heart failure leading to chronic renal dysfunction. However, the underlying mechanisms of CRS remains poorly defined and current therapeutic options are limited. Therefore, it is urgent to search for new treatment targets in CRS and develop more effective therapeutic approaches [5].

Fibroblast growth factor 23 (FGF23) was originally identified as a bone-derived hormone, but is also produced in the heart and kidneys [6, 7]. FGF23 is not just a regulator of mineral metabolism, since FGF23 deficiency has multiple effects, including vascular calcification, bone density decrease, growth retardation, and dramatic lifespan shortening [8]. However, it is unclear whether an excess of FGF23 is detrimental or beneficial. There is emerging evidence that FGF23 is closely associated with both cardiovascular and kidney diseases [9–14], indicating that FGF23 may play a role in the pathophysiology of CRS. Although the reason is still unclear, several reports revealed that FGF23 is a strong predictor of adverse cardiovascular outcomes in patients with chronic kidney disease (CKD) [15–19], suggesting a contribution of FGF23 to type 4 CRS (chronic renal dysfunction leading to cardiovascular dysfunction). However, it is completely unknown whether FGF23 is also involved in type 2 CRS (chronic heart failure leading to renal dysfunction). Considering the failing heart is a major source of circulating FGF23 [11], we postulated an increase of plasma FGF23 in patients with heart failure would contribute to kidney injury.

Downregulation or deficiency of Klotho, the co-receptor of FGF23, promotes oxidative stress and renal fibrosis [13, 14]. FGF23 preferentially stimulates left ventricular hypertrophy [10] and inhibition of Klotho augments myocardial fibrosis [13, 14], but whether FGF23 would promote renal fibrosis remains unclear. It was also reported that crosstalk occurred between the FGF23-Klotho axis and the fibrosis-promoting renin-angiotensin system [20]. Furthermore, a decrease of soluble Klotho in CKD was reported to be an important cause of uremic cardiomyopathy or to inhibit renal fibrosis in an FGF23-independent manner [21, 22], while FGF23 can induce myocardial hypertrophy independent of Klotho [10]. FGF receptor 4 (FGFR4) has been recently identified as a receptor of FGF23 in cardiomyocytes [23], while other FGF23 receptors (FGFR1, 2, and 4) express in the kidneys [24].

In chondrocytes, FGF23 was shown to activate fibrosis-related factors, such as matrix metalloproteinase-13, extracellular signal-regulated kinases (ERK), and the phosphatidylinositol 3-kinase (PI3K)/serine/threonine kinase (Akt) pathway by binding to FGFR1 independent of Klotho [25]. Recent evidence has suggested that FGF23 upregulation in the injured or diseased kidneys may augment myofibroblast activation and fibrogenesis by activation of TGF-β-related pathways [6, 26, 27]. Our recent study shows that FGF23 can not only be expressed in cardiomyocytes and fibroblasts, but also promote myocardial fibrosis in mice with MI or ischemia/reperfusion injury [28]. Therefore, we hypothesized that FGF23 may also play a role in renal fibrosis, either with Klotho or independent of Klotho. In this study, we generated a murine model of CRS by inducing MI for 12 weeks, and we used this model to investigate the potential role of FGF23 in renal fibrosis as well as its underlying mechanisms.

## RESULTS

### MI induces renal fibrosis and dysfunction

**Figure 1:**
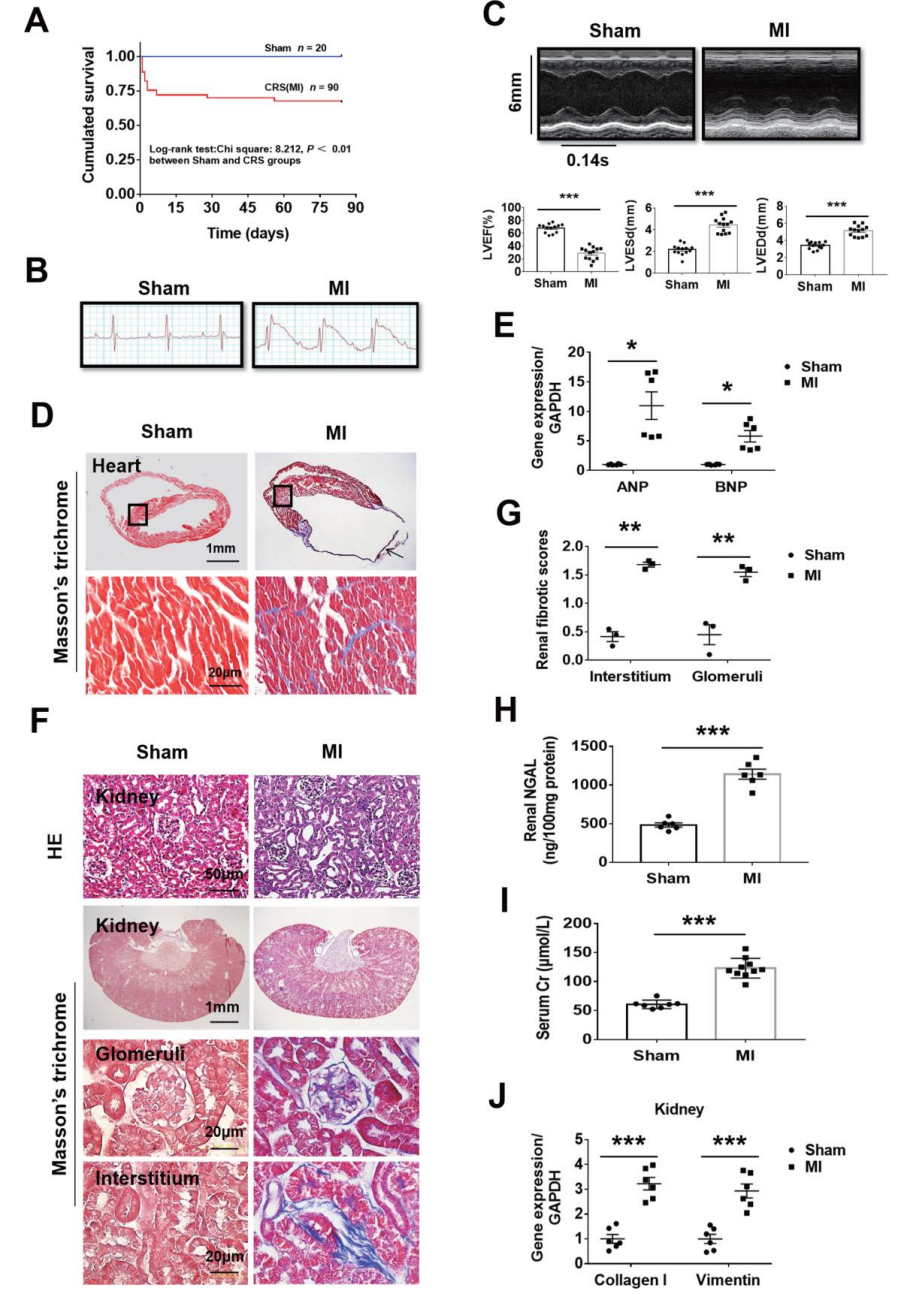
**Fibrosis and dysfunction of the heart and kidneys in mice with cardiorenal syndrome (CRS) after surgically induced myocardial infarction (MI) at 12 weeks.** (**A**) Survival rate for 12 weeks in Sham and CRS groups. (**B**) Representative electrocardiogram showing the ST-segment elevation in MI group. (**C**) Representative M-mode echocardiographic images. Left ventricular ejection fraction (LVEF), left ventricular end-systolic diameter (LVESd), and left ventricular end-diastolic diameter (LVEDd). ***P < 0.001 vs. sham mice, n = 13 per group. (**D**) Myocardial fibrosis detected by Masson’s trichrome staining in both the infarct zone (arrow) and the remote area (black frames). The lower panels are magnified views of the remote area (corresponding to black frames in the upper panels). (**E**) Quantitative real-time PCR for ANP and BNP mRNA in the heart. *P < 0.05 vs. sham mice, n = 6 per group. (**F**) Representative photomicrographs of hematoxylin-eosin staining (HE) and Masson-trichrome staining of kidney sections from sham and CRS groups show structural changes and fibrosis due to CRS. (**G**) Semi-quantitative analysis of renal fibrosis. **P < 0.01 vs. sham mice, n = 3 per group. (**H**) Renal NGAL level in renal tissue. (**I**) Serum creatinine level in mice measured by ELISA. For (**H** and **I**), ***P < 0.001 vs. sham mice, n = 6-10 per group. (**J**) Quantitative real-time PCR for Collagen I and Vimentin mRNA in the kidneys. ***P < 0.001 vs. sham mice, n = 6 per group. Data are means ± SE.

To observe whether MI can cause both heart failure and kidney damage, the mice were subjected to MI for 12 weeks. About 70% CRS mice could survive to 12 weeks and majority of the death happened in the first week after MI (Figure 1A). Autopsy found that the main causes of death were heart rupture and acute heart failure. Clear ST segment elevation after left coronary artery ligation was documented in all MI mice (Figure 1B). Echocardiography showed that MI mice had smaller left ventricular ejection fraction (LVEF), larger LV end-systolic diameter (LVESd) and LV end-diastolic diameter (LVEDd) than the sham ones (Figure 1C and Supplementary Table 1). Induction of MI for 12 weeks induced cardiac remodeling, myocardial fibrosis, cardiac dysfunction as well as kidney damage with glomerular and tubulointerstitial fibrosis accompanied by increased renal tissue neutrophil gelatinase-associated lipocalin (NGAL), elevated serum creatinine and upregulated the gene expression of Collagen I and Vimentin in the kidneys (Figure 1D–1J). Besides, in mice with MI for 12 weeks, serum 25 (OH) D3 levels were markedly increased, and serum phosphorus levels, systolic blood pressure (SBP) and renal mRNA expression of Napi2a, Napi2c and Cyp27b1 were significantly decreased, while no statistical differences were noted on kidney weight/body weight ratio (KW/BW) and renal mRNA expression of Cyp24a (Supplementary Figure 1A–1E). These findings indicated successful establishment of type 2 CRS model. We then investigated the effect of MI on fibrosis-associated signaling in the kidneys.

### Upregulation of FGF23 and FGFR4 in both the heart and kidneys and activation of fibrotic signaling in the kidneys by CRS

**Figure 2:**
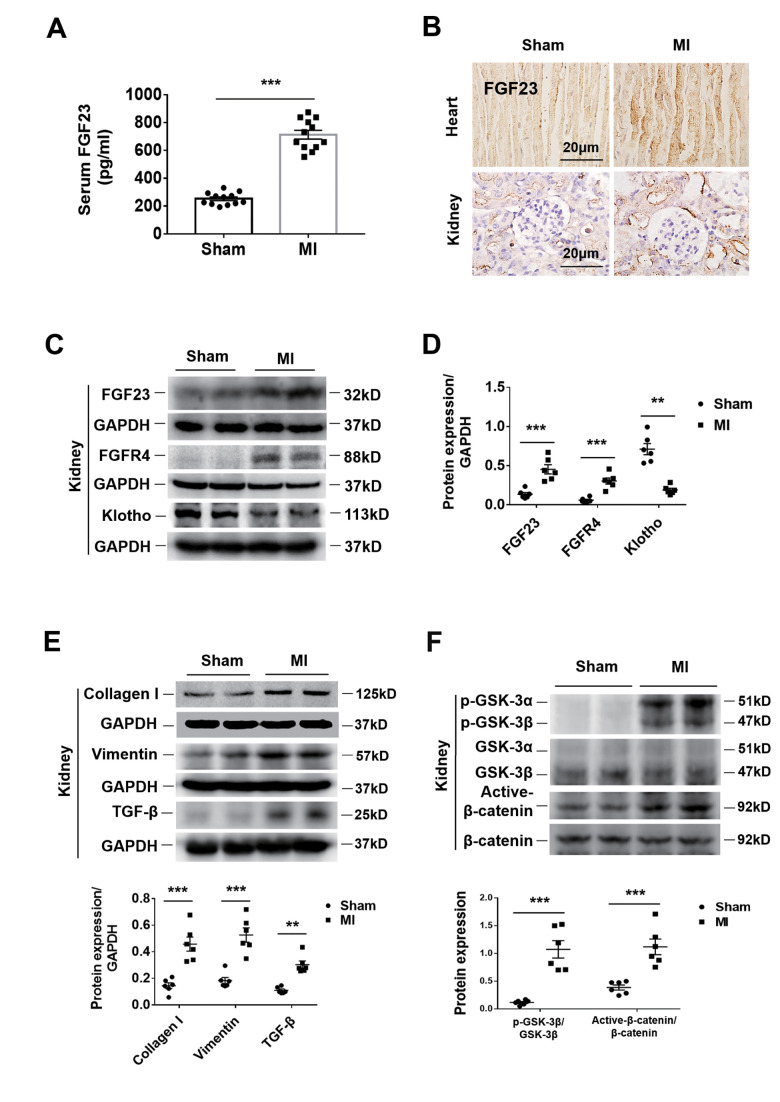
**Upregulation of FGF23 in the heart and kidneys and activation of fibrosis-related signaling pathways in the kidneys by cardiorenal syndrome (CRS) at 12 weeks after induction of MI.** (**A**) Serum FGF23 level measured by ELISA. ***P < 0.001 vs. sham mice, n = 12 per group. (**B**) Immunohistochemical staining shows enhanced FGF23 Protein expression in the myocardium and renal tubules of CRS mice compared with sham mice. (**C**) Western blot of FGF23, FGFR4 and Klotho in the kidneys of Sham and CRS mice. (**D**) Semi-quantitative assessment of FGF23, FGFR4 and Klotho. **P < 0.01, ***P < 0.001 vs. sham mice, n = 6 per group. (**E**) Western blot of collagen I, vimentin and TGF-β. **P < 0.01, ***P < 0.001 vs. sham mice, n = 6 per group. (**F**) Western blotting reveals significant upregulation of p-GSK-3β and active-β-catenin protein expression in the kidneys of CRS mice. ***P < 0.001 vs. sham mice, n = 6 per group. Data are means ± SE.

The serum FGF23 concentration was significantly higher in CRS mice than in sham ones (Figure 2A). Immunohistochemistry showed an increase of FGF23 in both the heart and kidneys of CRS mice (Figure 2B). Because FGFR4 has been reported to be a receptor of FGF23 that exerts biological activity independent of its co-receptor Klotho in cardiomyocytes [23], we further investigated FGF23 and FGFR4 gene expression in the heart and kidneys. The results of qPCR showed that cardiac FGF23, cardiac FGFR4 and renal FGFR4 were significantly upregulated, whereas renal FGF23 was not (Supplementary Figures 2A, 2B, 3F). Consistently, FGFR4 protein expression was also found to be upregulated in the kidneys of CRS mice (Figure 2C, 2D). Furthermore, renal immunostaining for FGFR4 was enhanced in CRS mice and localized in both the glomeruli and tubules (Supplementary Figure 2C). In CRS mice, the results of western blot showed that the expression of FGF23 was upregulated in the kidneys, while the expression of Klotho was significantly downregulated in the kidneys (Figure 2C, 2D). Immunohistochemistry showed that Klotho expressed in renal tubules rather than the glomeruli of sham mice, and its expression was significantly decreased in the renal tubules of CRS mice (Supplementary Figure 2D).

In CRS mice, collagen I, vimentin and Transforming growth factor (TGF-β) were upregulated in the kidneys (Figure 2E). Renal expression of phosphorylated glycogen synthase kinase 3 (p-GSK-3β) and active-β-catenin proteins was also significantly increased (Figure 2F). In addition, the renal mitogen-activated protein kinases (MAPK) pathway was activated simultaneously, but had no effect on the calcineurin-NFAT (nuclear factor of activated T cells protein) pathway (Supplementary Figure 2E). However, the results in cultured NRK-49F cells showed that FGF23 stimulation had no significant effect on MAPK or calcineurin-NFAT pathways (Supplementary Figure 2F). Therefore, we focused our attention on the β-catenin signaling pathway.

### Myocardial overexpression of FGF23 promotes myocardial fibrosis and upregulation of FGF23 in both the heart and kidneys of CRS mice

**Figure 3:**
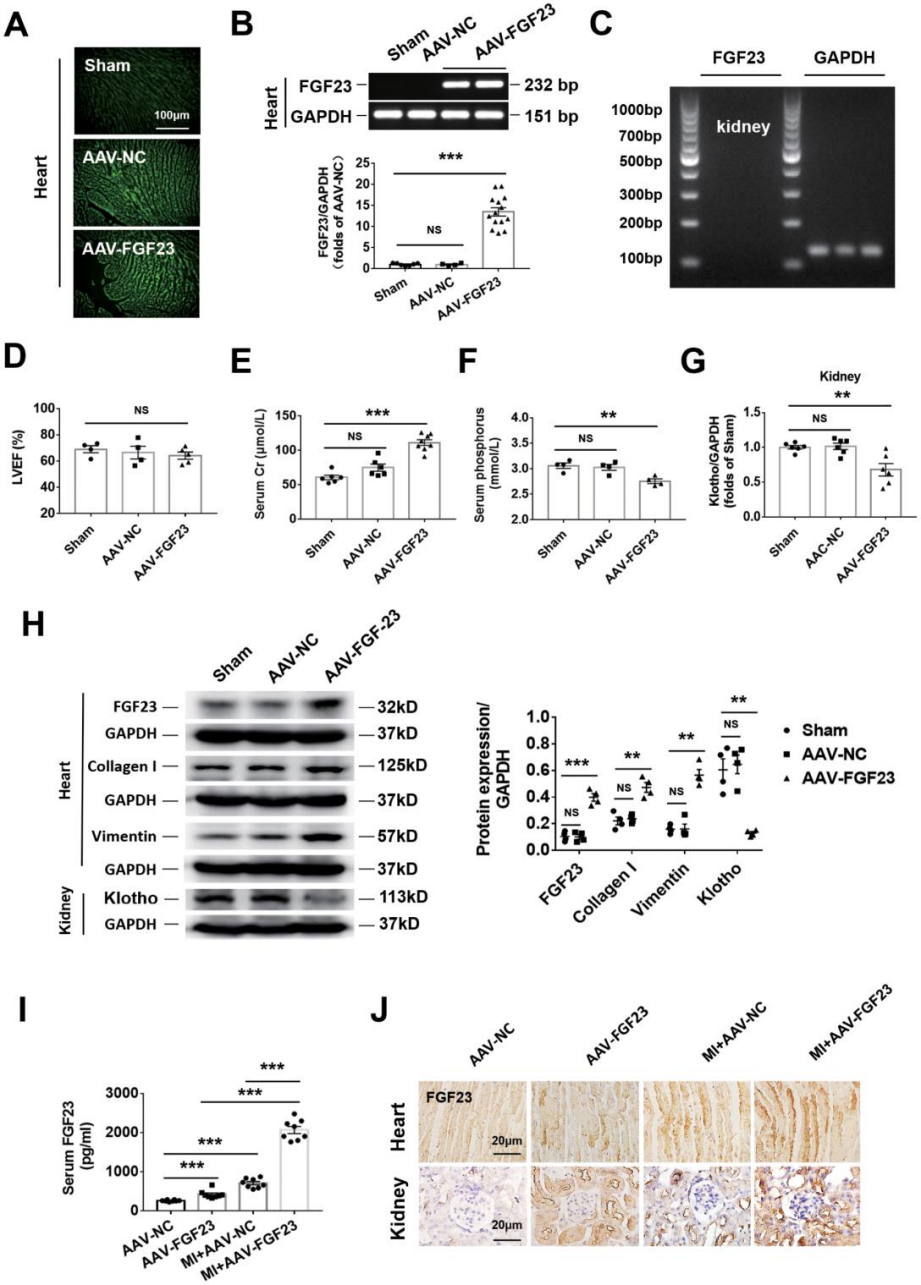
**Cardiac overexpression of FGF23 enhanced cardiac fibrosis and renal dysfunction.** (**A**) The results of frozen sections showed that adeno-associated virus (AAV) carrying the FGF23 plasmid or negative control (NC) successfully infected the heart. (**B**) Confirmation of FGF23 overexpression in mouse hearts. Myocardial FGF23 mRNA was measured by routine and real-time PCR 4 weeks later. ***P < 0.001. NS, no statistical significance. n = 4-14 per group. (**C**) Expression of FGF23 mRNA was not detected in the kidneys. (**D**) Left ventricular ejection fraction (LVEF) showed no statistical significance between the groups. Serum creatinine (**E**) and serum phosphorus (**F**) levels were measured by ELISA. **P < 0.01, ***P < 0.001. NS, no statistical significance, n = 4-8 per group. (**G**) Routine PCR and quantitative real-time PCR for Klotho mRNA in the kidneys. **P < 0.01 vs. sham group, n = 6 per group, respectively. (**H**) Western blot of FGF23, collagen I and Klotho in the heart and kidneys. **P < 0.01, ***P < 0.001 vs. sham group, n = 4 per group. (**I**) Serum FGF23 was measured by ELISA. ***P < 0.001. n = 8 per group. (**J**) Immunohistochemical detection of FGF23 expression in the heart and kidneys of mice with or without cardiorenal syndrome and injection of AAV-NC or AAV-FGF23. Data are means ± SE.

Intra-myocardial injection of adeno-associated virus containing FGF23 (AAV-FGF23) was performed in 6-week-old mice to induce cardiac overexpression of FGF23. Four weeks after injection, the results of frozen cardiac sections showed that the AAV-FGF23 successfully infected the myocardium (Figure 3A). Furthermore, the results of real-time PCR indicated that there was an approximately 15-fold increase of FGF23 expression compared with that in the negative control group (Figure 3B). Due to the very low gene expression level of FGF23 at baseline in the heart and kidneys (Figure 3B and C), we used pharmaceutical inhibitory approaches rather than gene silencing to test the effect of function loss. After myocardial overexpression of FGF23 for 16 weeks, echocardiography showed no effect on LVEF (Figure 3D), while ELISA results showed a significant increase and decrease on serum creatinine and phosphorus concentrations, respectively (Figure 3E, 3F). Overexpression of FGF23 exerted no significant influence on SBP and renal gene expression of Cyp24a, but significantly increased serum 25 (OH) D3 and decreased serum phosphorus and renal gene expression of Napi2a, Napi2c and Cyp27b1 (Supplementary Figure 1A–1E). Real-time PCR showed that Klotho's mRNA was downregulated in the kidneys (Figure 3G). The results of western blot indicated that not only FGF23 was upregulated, but also fibrosis-associated protein collagen I and vimentin were upregulated in the heart after myocardial overexpression of FGF23. However, FGF23 co-receptor klotho was downregulated in the kidneys (Figure 3H). In addition, overexpression of FGF23 in the myocardium also induced cardiac hypertrophy, increased myocardial cell area and upregulated the expression of hypertrophic related genes (Supplementary Figure 3A–3E). At 12 weeks after MI, serum FGF23 concentration was significant higher in the AAV-FGF23 and MI+AAV-NC groups than the AAV-NC group. In CRS mice, myocardial overexpression of FGF23 was associated with a significant further increase of the circulating FGF23 concentration compared with the AAV-FGF23 and MI+AAV-NC groups (Figure 3I). Similar results were obtained for FGF23 by immunohistochemistry analysis in both the heart and kidneys, with renal FGF23 expression being localized in the tubules (Figure 3J). However, there was no change in the expression level of FGF23 gene in the kidneys of MI and MI+FGF23 groups (Supplementary Figure 3F).

### Myocardial overexpression of FGF23 promotes renal fibrosis and activates fibrosis-related signaling

**Figure 4:**
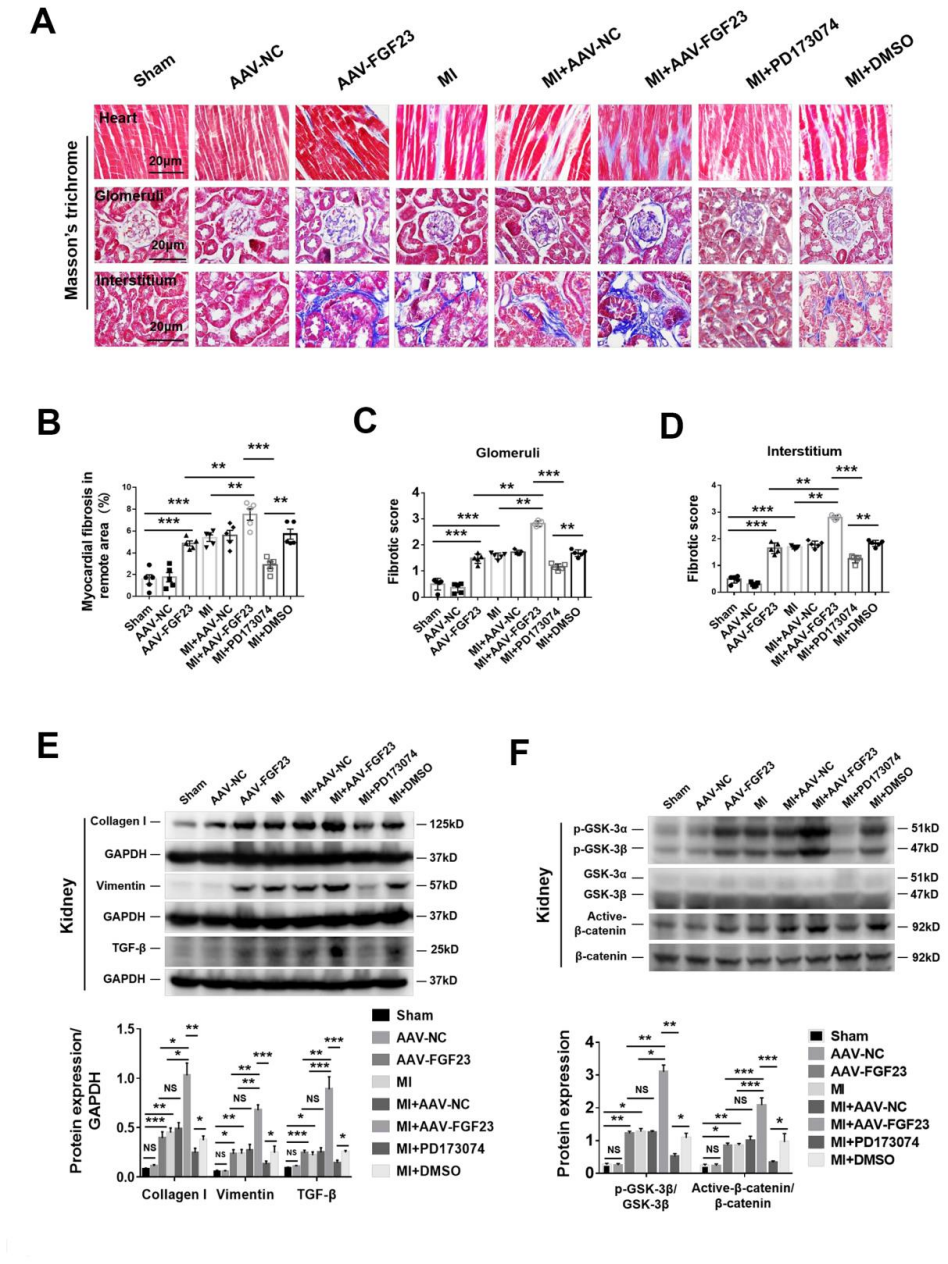
**FGF23 overexpression further enhanced cardiac and renal fibrosis, and promoted activation of fibrosis-related signaling pathways in the kidneys of mice with cardiorenal syndrome (CRS).** (**A**) Representative photomicrographs of myocardial and renal fibrosis detected by Masson’s trichrome stain in sham, AAV-NC and AAV-FGF23 mice or CRS mice injected with AAV-NC or AAV-FGF23, or treated with PD173074 or DMSO. (**B**–**D**) Semi-quantitative assessment of myocardial, renal glomerular and interstitial fibrosis. (**B**–**D**) **P < 0.01, ***P < 0.001. n = 5 per group. (**E**) Western blot of collagen I, vimentin and TGF-β. (**F**) Western blot of p-GSK-3β and active-β-catenin. For (**E** and **F**), *P < 0.05. **P < 0.01, ***P < 0.001. NS, no statistical significance, n = 3 per group. Data are means ± SE.

As expected, cardiac fibrosis, glomerular and renal interstitial fibrosis were markedly enhanced in CRS mice by cardiac overexpression of FGF23 (Figure 4A–4D). Considering that FGF23 can bind with low affinity to FGFR1c, FGFR3c and FGFR4 [29], we used a pan-FGFR inhibitor PD173074 to further test the effect of FGF23 on cardio-renal fibrosis. We noted that the fibrosis was apparently suppressed by treatment with PD173074 (Figure 4A–4D). Protein levels of collagen I, vimentin, TGF-β, p-GSK-3β and active-β-catenin were significantly upregulated by myocardial overexpression of FGF23 in CRS mice compared with the MI alone or sham ones, which were suppressed by PD173074 treatment (Figure 4E, 4F). Immunohistochemical analysis also revealed an increase of TGF-β in the kidneys of FGF23-overexpressing MI mice, which was suppressed by co-treatment with PD173074 (Supplementary Figure 4A, 4B). In contrast, renal expression of Klotho was reduced in CRS mice, but overexpression of FGF23 or treatment with PD173074 did not further change Klotho expression (Supplementary Figure 4A–4C). Similar results were obtained for Klotho by Western blot analysis (Supplementary Figure 4D, 4E). FGFR4 antagonist BLU9931 also significantly inhibited MI-induced renal fibrosis (Supplementary Figure 5A–5C).

**Figure 5:**
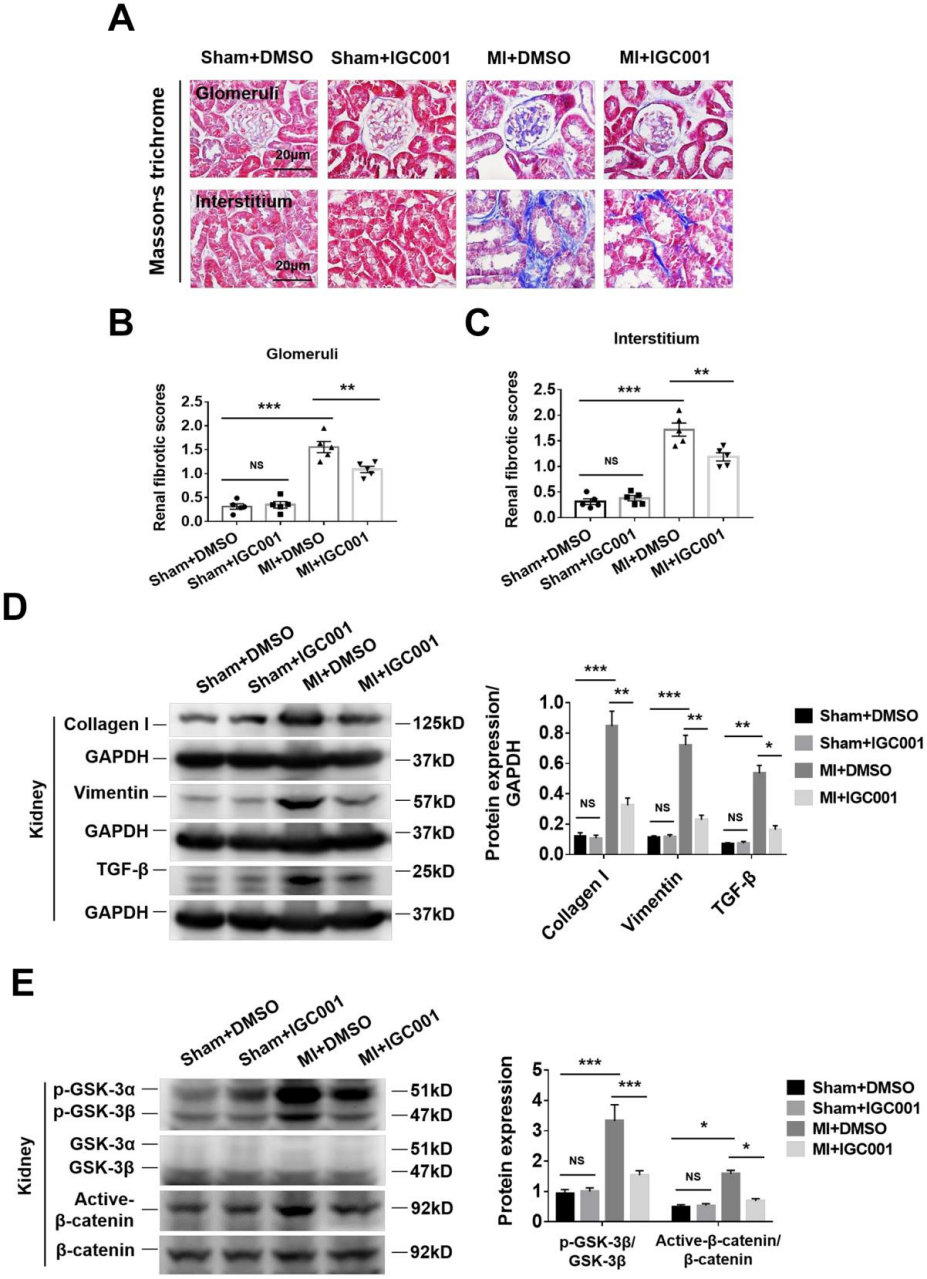
**Activation of fibrosis-related signaling pathways can be prohibited by β-catenin inhibitor IGC001 (5 mg/kg/d) in the kidneys at 12 weeks after MI.** (**A**) Representative photomicrographs of renal fibrosis detected by Masson’s trichrome stain in sham mice or CRS mice treatment with IGC001 or DMSO. Semi-quantitative assessment of glomerular fibrosis (**B**) and interstitial fibrosis (**C**). **P < 0.01, ***P < 0.001. NS, no statistical significance, n = 5 per group. (**D**) Western blot of collagen I, vimentin and TGF- β. (**E**) Western blot of p-GSK-3β and active-β-catenin. *P < 0.05. **P < 0.01, ***P < 0.001. NS, no statistical significance, n = 3 per group. Data are means ± SE.

### Inhibition of β-catenin reduces renal fibrosis in CRS mice

Renal glomerular and renal interstitial fibrosis were significantly enhanced in CRS mice than in sham mice, while treatment with β-catenin antagonist IGC001 suppressed this effect (Figure 5A–5C). Western blot analysis demonstrated that expression of collagen I, vimentin and TGF-β was significantly elevated in CRS mice compared with sham mice, and the upregulation of these proteins was suppressed by IGC001 treatment (Figure 5D). Similar results were obtained for both p-GSK-3β and active-β-catenin (Figure 5E).

### Profibrotic effect of FGF23 is associated with FGFR4 and β-catenin in cultured renal fibrotic cells

**Figure 6:**
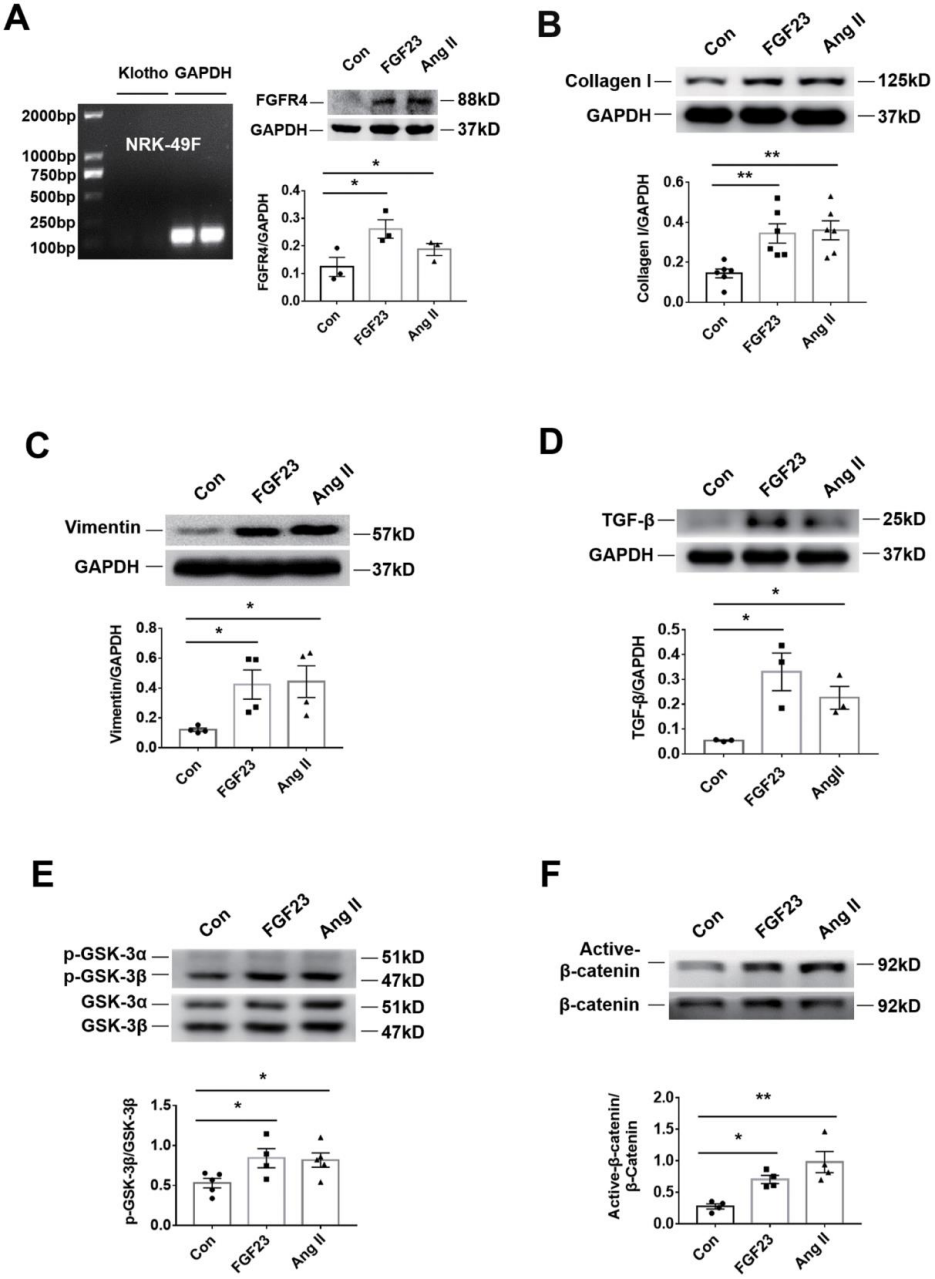
**Recombinant FGF23 promotes activation of fibrosis-related signaling pathways in NRK-49F cultured kidney fibroblasts.** (**A**) Expression of Klotho mRNA was not detected. Western blotting showed that FGFR4 protein expression was upregulated by treatment with recombinant FGF23 (100 ng/mL) or angiotensin II (Ang II, 1 μM). (**B**) Western blot of collagen I. (**C**) Western blot of vimentin. (**D**) Western blot of TGF-β. (**E**) Western blot of p-GSK-3β. (**F**) Western blot of active-β-catenin. Con, control; FGF23, fibroblast growth factor 23; Ang II, angiotensin II. *P < 0.05, **P < 0.01, n = 3-6 per group. Data are means ± SE.

**Figure 7:**
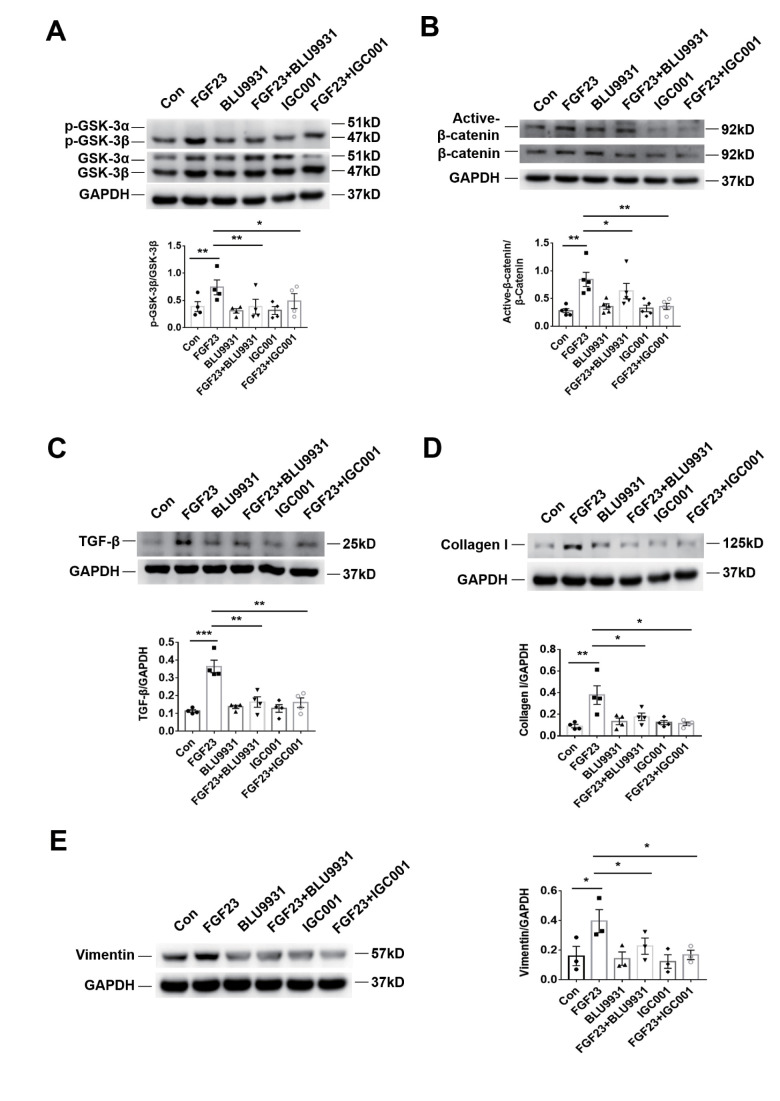
**Profibrotic effect of FGF23 can be inhibited by treatment with FGFR4 inhibitor BLU9931 or β-catenin inhibitor IGC001.** (**A**) Western blot of p-GSK-3β. (**B**) Western blot of active-β-catenin. (**C**) Western blot of TGF-β. (**D**) Western blot of collagen I. These representative blots were performed in the same gel as (**A**), so that both panels shared the same loading controls. (**E**) Western blot of vimentin. BLU9931, 100 nM; IGC001, 5 μM. *P < 0.05, **P < 0.01, n = 3-5 per group. Data are means ± SE.

In the rat renal fibroblast cell line NRK-49F, expression of Klotho mRNA was undetectable, but FGFR4 protein was detected (Figure 6A). Western blot analysis demonstrated that incubation with recombinant FGF23 significantly upregulated collagen I, vimentin, TGF-β, p-GSK-3β, and active-β-catenin proteins, similar to the pro-fibrotic agent angiotensin II (Figure 6B–6F). Recombinant FGF23 also upregulated the expression of TGF-β and collagen I mRNA in cultured NRK-49F cells (Supplementary Figure 6A, 6B). In addition, FGF23 had a certain proliferative effect on fibroblasts (Supplementary Figure 6C). To investigate the role of FGFR4 and β-catenin in the pro-fibrosis of FGF23, we co-stimulated NRK-49F cells with recombinant FGF23 and FGFR4 inhibitor BLU9931, or β-catenin inhibitor IGC001. Western blot results showed that recombinant FGF23 significantly upregulated p-GSK-3β, active-β-catenin, TGF-β, collagen I and vimentin proteins, and the expression of these proteins was apparently suppressed by treatment with BLU9931 or IGC001 (Figure 7A–7E).

## DISCUSSION

FGF23 plays an essential role in phosphate homeostasis and growth. FGF23-FGFR/α-Klotho complex regulates the metabolism of calcium, phosphorus, and vitamin D. In mice, the loss of FGF23 or Klotho retards growth and shortens the lifespan [8]. Somewhat surprisingly, transgenic mice for wild-type human FGF-23 do not gain a longer lifespan and still show growth retardation [30]. These findings suggest that the optimal FGF23 level is essential for maintaining normal life. There is emerging clinical evidence that high FGF23 level is associated with an increased risk of the progression of CKD, cardiovascular events, and death [9, 12, 17, 31, 32]. Therefore, we investigated whether an excess of FGF23 had an influence on renal fibrosis in mice with type 2 CRS, and we obtained evidence that the increase of circulating FGF23 caused by MI or overexpression of FGF23 can promote renal fibrosis. However, we also found that the decrease of Klotho protein in kidney. Whether the decrease of Klotho protein in the kidney is another cause of renal fibrosis or only a concomitant result needs more intervention experiments to determine.

Although a previous research observed an upregulation of both osteocytic and myocytic FGF23 expression in post-MI mice [33], recent evidence indicates that conditional ablation of FGF23 in bone cells only resulted in moderate reduction in circulating FGF23 levels [34, 35] and cardiac fibroblasts (not the bone or macrophages) were reported to be the only source of local FGF23 production after MI [7]. In this study, we found that not only cardiac FGF23 but also the expression levels of FGF23 in kidney and circulating were increased in MI mice with heart failure, in good agreement with previous reports [11, 33]. Fajol et al. recently reported that FGF23 secretion could be stimulated by the sympathetic nervous system, and enhanced FGF23 production in mice could be normalized by β-blocker treatment [36]. It is well known that the sympathetic activity is increased by MI or heart failure, which may be one of the reasons for elevation of the plasma FGF23 concentration in our mice with MI and heart failure. There is clinical evidence that an elevated FGF23 level is associated with cardiovascular death and all-cause mortality, while reduction of serum FGF23 by treatment with cinacalcet is associated with lower rates of cardiovascular death and major cardiovascular events [31]. Experimental evidence from other authors and our laboratory has shown that excess FGF23 promotes cardiac remodeling by increasing myocardial hypertrophy and fibrosis [10, 28]. However, before this study it was unclear whether excess FGF23 production in the heart is also detrimental for the kidneys.

Why was renal FGF23 increased in the kidneys of CRS mice with myocardial overexpression of FGF23? As a secreted protein, FGF23 can be produced from various organs such as bone, heart and kidneys and can also enter into the blood circulation to reach distant organs [27, 37]. Our results show that induction MI for 12 weeks increased the content of FGF23 protein in the heart, serum and kidneys. Considering that gene expression level of FGF23 in the kidneys was not significantly changed, the slight increase of FGF23 protein in the kidneys may come from the infarct heart. Consistent with this model, myocardial overexpression of FGF23 not only upregulated FGF23 protein expression in the heart, but also increased FGF23 protein levels in the circulation and kidneys without changing kidney FGF23 mRNA levels. As shown in Figure 3I, we noted a synergistic effect rather than an additive effect between MI and FGF23 overexpression, suggesting a positive feedback may exist between cardiac dysfunction and circulatory FGF23 levels. Our previous study showed that FGF23 overexpression could worsen heart failure [28], while high levels of FGF23 were significantly associated with the severity of heart failure [38]. Although both heart and kidney can produce FGF23, our results demonstrated that renal FGF23 gene was not changed in response to chronic MI or myocardial overexpression of FGF23, supporting that the heart is critical for FGF23 production in type 2 CRS. Considering that FGF23 can bind with low affinity to different FGFRs [29], which is enhanced by a cofactor Klotho in its target organs such as the kidneys and the parathyroid gland [29], it is reasonable that myocardial overexpression of FGF23 increased FGF23 protein production in the heart and then traveled to the kidney through blood circulation and bound with FGFRs and Klotho, eventually increased the renal levels of FGF23 accordingly.

Our finding that myocardial overexpression FGF23 in MI mice decreased serum phosphorus level and worsened renal function could suggest the hypothesis that phosphate supplementation could alleviate the severity of CRS. However, it is known that FGF23 prevents the increase of serum phosphate in early stage of CKD, whereas in end-stage FGF23 fails to maintain phosphate homeostasis. Both hyperphosphatemia and excessive FGF23 can promote the development of hypertension, vascular calcification, and left ventricular hypertrophy [39]. Taken together, it is possible that phosphate supplement wouldn’t be able to alleviate CRS. In contrast, there is evidence that direct targeting of phosphate and FGF receptors can prevent toxicity of FGF23 and hyperphosphatemia in CKD patients [23]. In addition, we noticed that the degree of renal fibrosis in the MI mice was markedly aggravated by a moderate increase of FGF23 protein through FGF23 gene overexpression and significantly alleviated by FGF23 receptor antagonist PD173074, suggesting a causative relation between FGF23 and renal fibrosis. Besides, our previous report has demonstrated that intra-myocardial injection of AAV-FGF23 in mice significantly worsened diastolic dysfunction and promoted myocardial fibrosis [28], which may contribute to renal damage in CRS.

Because of the limited number of organs that co-express the FGFR/α-Klotho complex, it is also possible that the influence of elevated circulating FGF23 levels is directly mediated by off-target effects due to FGF23 activating FGF receptors independent of Klotho. The present study revealed that FGF23 promoted both glomerular and renal tubular fibrosis *in vivo* and increased the expression of fibrosis-related proteins of renal fibroblasts, which does not express klotho, suggesting that Klotho-independent actions of FGF23 could be important. In the kidneys, Klotho is not expressed in the glomeruli or by fibroblasts, and is only expressed in the tubular epithelium. Our findings indicate that FGF23 promotes renal fibrosis may not depend on Klotho. The ectodomain of Klotho is released into the circulation and exerts activity on other tissues or organs. In our mouse model of type 2 CRS, downregulation of renal Klotho expression may have contributed to fibrosis of both the glomeruli and tubules. In consistence with our previous study [40], heart failure could cause renal fibrosis accompanied by Klotho depletion and β-catenin activation in the kidney. One of the reasons for Klotho downregulation in type 2 CRS maybe the increase of circulatory tumor necrosis factor-α induced by heart failure [40]. Similarly, in several mouse models (remnant kidney, adriamycin nephropathy, and unilateral ureteral obstruction), Zhou et al. demonstrated a significant downregulation of renal Klotho expression and showed that Klotho overexpression ameliorated renal fibrosis [14, 41].

Why does excess FGF23 promote fibrosis? Previous studies have provided a few clues. Bianchi et al. found that FGF23 drove expression of matrix metalloprotein-13 and type X collagen alpha 1 chain in human osteoarthritic chondrocytes in a Klotho-independent manner [25], while Faul et al. demonstrated that FGF23 induced myocardial hypertrophy by activation of the calcineurin-NFAT signaling pathway [10]. It is thought that MMPs, calcineurin, and collagens are all closely associated with fibrosis [42, 43]. Dai et al. found that FGF23 decreased the renal expression of α-Klotho and angiotensin-converting enzyme 2 and that FGF23 activated the lipocalin 2, TGF-β, and tumor necrosis factor α signaling pathways [44]. It is well known that ACE2, TGF-β, and TNF-α are important in the pathogenesis of fibrosis. Evidence indicates that activation of the ACE2-Ang-(1-7)-Mas axis has a counter-regulatory effect on the Ang II type 1 receptor activation and inhibits fibrogenesis [45]. In addition, anti-TNF-α therapy was reported to improve cardiac fibrosis in rats with experimental diabetic cardiomyopathy and reduce renal fibrosis in rats with renal failure [46, 47]. Furthermore, TGF-β is reported to be the master regulator that drives fibrosis in chronic kidney disease [48].

Signaling through the FGF family is involved in the key regulatory pathways of fibrosis. Olauson et al. proposed that FGF23 excess and Klotho deficiency were interdependent, with FGF23 preferentially stimulating left ventricular hypertrophy and loss of Klotho augmenting fibrosis [13]. In this study, we observed that FGF23 promoted renal fibrosis in CRS mice with upregulation of TGF-β expression. It has been reported that TGF-β is important for the accumulation of extracellular matrix components in the glomeruli (glomerular fibrosis) and tubulointerstitial fibrosis [49]. In CRS mice, we found that cardiac overexpression of FGF23 enhanced TGF-β expression in renal tubules along with an increase of tubulointerstitial fibrosis. TGF-β has been shown to mediate fibroblast proliferation, as well as tubular and fibroblast production of extracellular matrix, leading to interstitial fibrosis [49]. It is widely accepted that resident fibroblasts are the major source of collagen-producing myofibroblasts involved in tubulointerstitial fibrosis. Therefore, we evaluated the fibrogenic effect of FGF23 on fibroblasts using NRK-49F cells, and found that FGF23 promoted the activation of fibrosis-related signaling pathways of these cells as well as increased expression of collagen and vimentin.

Consistent with our previous study [28], we confirmed that FGF23 upregulated active-β-catenin expression in the kidneys of CRS mice and in a renal fibroblast cell line. Katoh reported that FGF20 was a target of the WNT-β-catenin signaling pathway and cross-talk between the WNT and FGF signaling pathways potentiated the β-catenin and NFAT signaling cascades [50]. Interestingly, FGF23 has been shown to activate β-catenin and NFAT signaling [10, 28], both of which are closely associated with fibrosis. It is known that cross-talk exists between Wnt/β-catenin and TGF-β signaling, which cooperate in the process of fibrosis, and TGF-β induces the expression of Wnt/β-catenin superfamily members and vice versa [14, 51–54]. Importantly, this cross-talk is complex and context-dependent, and may promote fibrogenesis through the co-regulation of fibrogenic gene targets [51, 53, 54]. In cultured NRK-52E cells, FGF23 was reported to promote the deposition of extracellular matrix induced by TGFβ1, and aggravate the degree of renal interstitial fibrosis in unilateral ureteral occlusion model, which was associated with the activation of β-catenin signaling pathway [37]. We found upregulation of renal active-β-catenin expression in CRS mice with myocardial overexpression of FGF23, which was accompanied by upregulation of TGF-β and deposition of collagen I. FGF23 is known to inhibit renal synthesis of active vitamin D, while treatment with vitamin D analogs can suppress upregulation of active-β-catenin, TGF-β1, and collagens in chronic kidney disease [55], supporting our finding that excess FGF23 upregulates active-β-catenin, TGF-β1, and collagens in CRS mice. In addition, we also found that the up-regulation of these proteins could be reversed by a β-catenin inhibitor IGC001, further suggesting the important role of β-catenin signaling pathway in the process of FGF23 pro-fibrosis. Our previous reports showed that β-catenin played important role in cardiac or renal fibrosis [28, 40, 56] and FGF23 promoted myocardial fibrosis in post-MI mice mediated by activation of β-catenin [28]. Our finding that FGF23 promoted fibrogenesis in cultured NRK-49F cells is paradoxical to the findings by Smith ER et al [6]. However, a recent study by Wei et al demonstrated that TGF-β1 treatment in NRK-49F cells can promote fibrogenesis [57], lending to support our *in vitro* finding that FGF23 promoted fibrogenesis by upregulation of TGF-β.

The role of GSK-3β in fibrosis is controversial and seems to be context-dependent [58–60]. We demonstrated that excess FGF23 increased renal p-GSK-3β expression in CRS mice and renal fibroblasts. Singh et al. reported that GSK-3β could promote ischemia/reperfusion-induced renal fibrosis by activation of TGF-β signaling [59]. In addition, Bahammam et al. reported that inhibition of GSK-3β in gingival fibroblasts attenuated the expression of connective tissue growth factor, which mediates TGF-β-induced fibrosis [60]. Ahmad et al. reported that cardiomyocyte-specific GSK3α deletion attenuated post-infarction cardiac remodeling and heart failure [61]. Additionally, Chen et al. reported that increase of GSK-3β in periostin knockout mice impaired post-MI regeneration of the myocardium, while SB216763, a pan inhibitor of both GSK-3α and GSK-3β, improved myocyte regeneration and attenuated cardiac remodeling in post infarct mice deficiency of periostin [62].

Previous studies demonstrated that FGF23 inhibited parathyroid hormone secretion through MAPK pathway [63] or induced myocardial hypertrophy mediated by activation of PLCγ-calcineurin-NFAT pathway [23]. Our results showed that the MAPK pathway was significantly activated in the kidneys of CRS mice, but there was no significant change on the calcineurin/NFAT pathway, while in cultured fibroblasts of NRK-49F cells, we noted that FGF23 exerted no significant influence on either MAPK or calcineurin-NFAT pathway, partially in agreement with a previous study showing that FGF23 cannot activate MAPK signaling pathway in the absent of Klotho [64].

The role of FGFR4 in the process of renal fibrosis is not fully understood. A recent study has shown that in the absence of α-Klotho, FGF23 aggravated pro-fibrotic signaling cascades in injury-primed renal fibroblasts via activation of FGFR4 and upregulation of transient receptor potential cation channel 6, a calcium transporter [65]. Consistently, our findings in this study have shown that inhibition of FGF23 receptor could significantly inhibit the profibrotic effect of FGF23 in kidneys of CRS mice. Although it remains unclear how much contribution of FGFR4 to renal fibrosis, our *in vitro* results confirmed that inhibition of FGFR4 significantly inhibited the profibrotic effect of FGF23, suggesting that FGF23-FGFR4 axis plays an important role in renal fibrosis. In term of the role of Klotho in CRS, further studies using Klotho overexpression and silencing approaches would be helpful. The increase of FGFR4 expression in the heart of the CRS mice maybe attributable to the compensatory myocardial hypertrophy induced by MI and the upregulation of its ligand FGF23, and the similar phenomenon has been reported from both experimental and clinical investigations [66, 67]. It was reported that FGF23 binds with low affinity to FGFR1c, FGFR3c and FGFR4 [44, 67], and activation of FGFR1 or FGFR4 by FGF23 is associated with organ remodeling [6, 10, 23]. In this study, we found that activation of FGF23/FGFR4 signaling pathway promotes renal fibrosis, which was mitigated by either PD173074 or BLU9931, a FGFRs non-specific inhibitor or a FGFR4 specific inhibitor, a loss of function experiments of FGF23 (such as knockdown of FGF23 in heart) would strengthen this conclusion. We should speculate that other factors may also contribute to the renal fibrosis in type 2 CRS. Coincidently, a recent report shows that activation of the renin-angiotensin system and tumor necrosis factor-α in pressure overloaded heart also contribute to the renal injury [40].

**Figure 8:**
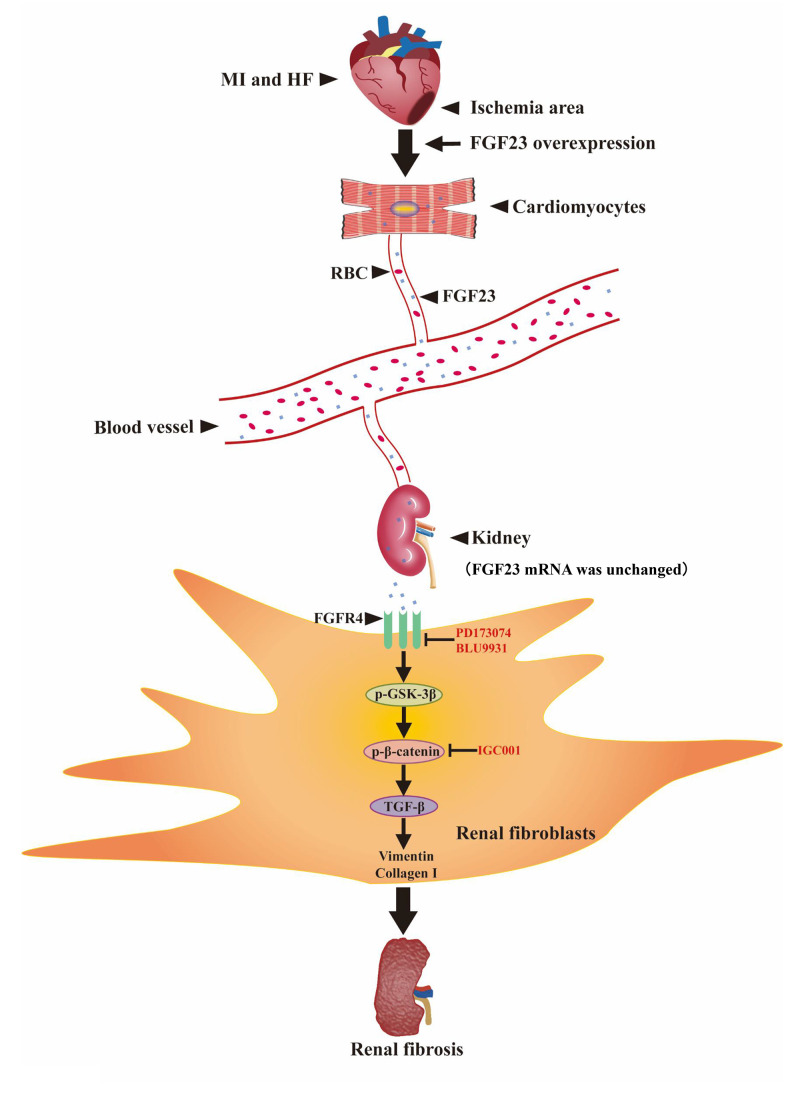
**Schematic illustration the contribution of FGF23 induced by post-infarct heart failure to promoting renal fibrosis.** Myocardial infarction and heart failure up-regulates FGF23 in cardiomyocytes and increases FGF23 protein level in the kidneys through blood circulation. FGF23 binds FGFR4 in renal fibroblasts and then activates p-GSK3β/p-β-catenin/TGF-β/Vimentin/Collagen I signaling pathway to promote renal fibrosis. Intervention with FGF23 overexpression or FGFR antagonist or β-catenin inhibitor can change the degree of renal fibrosis. MI, myocardial infarction; HF, heart failure; FGF23, fibroblast growth factor 23; RBC, red blood cell; FGFR4, fibroblast growth factor receptor 4; p-GSK3β, phosphorylated glycogen synthase kinase-3 beta; p-β-catenin, phosphorylated beta-catenin; TGF-β, transforming growth factor-beta; PD173074, FGF receptor antagonist; BLU9931: FGFR4 antagonist; IGC001, β-catenin antagonist; ↑, activation; Т, inhibition.

In summary, our present findings indicate that excessive FGF23 can promote renal fibrosis in type 2 CRS mice by binding to FGFR4 and activating β-catenin signaling pathway (Figure 8).

## MATERIALS AND METHODS

The study was reviewed and approved by the Institutional Review Board of Southern Medical University. All study procedures complied with the Institutional Guidelines for Animal Research and conformed to the Guide for the Care and Use of Laboratory Animals published by the United States National Institutes of Health (revised 1996).

### CRS model

Ten-week-old male C57BL/6 mice (provided by our university Experimental Animals Center) were anesthetized by inhalation of 2% isoflurane, and MI was induced by permanent ligation of the left coronary artery as described elsewhere [68]. Some mice were injected with adeno-associated virus (AAV carrying the FGF23 gene or a negative control (NC) into the myocardium at 4 weeks before MI. Some mice were intraperitoneally given a pan-FGF receptor antagonist PD173074 (10 mg/kg/d, Sigma-Aldrich, Darmstadt, Germany) [prepared in 12.5% cremophor EL containing 2.5% dimethyl sulfoxide (DMSO)] or orally administered a β-catenin antagonist IGC001 (5 mg/kg/d, Selleck, TX, USA) (dissolved in 2% DMSO + 50% polyethylene glycol 300 + 5% Tween 80 + ddH2O) or initiated the same volume of DMSO at 4 weeks after MI and persisted for 8 weeks. After 12 weeks of observation, mice were sacrificed with an overdose of pentobarbital sodium (150 mg/kg intraperitoneally) and cervical dislocation. The hearts and kidneys were harvested and weighed for further analysis. For histological examinations, hearts and kidneys were fixed in 4% paraformaldehyde, whereas for molecular analysis the hearts and kidneys were snap-frozen in liquid nitrogen and stored at -80° C until used.

### Cell culture

A normal rat kidney fibroblast cell line NRK-49F was cultured for 24 h in the presence/absence of 1μM angiotensin II (Ang II; Sigma-Aldrich, Darmstadt, Germany) or 100 ng/mL recombinant FGF23 (2629-FG-025, R&D Systems, MN, USA) or 100nM BLU9931 (a specific FGFR4 antagonist, Selleck, TX, USA) or 10μM IGC001. The concentration of recommended FGF23 protein in our study was determined according to previous reports [69] and our preliminary experiments. Then the cells were harvested at the indicated times for real-time PCR or western blotting.

### Measurement of FGF23 and assessment of renal function

At 12 weeks after starting the induction of CRS, blood samples were collected from the right ventricle of euthanized mice. The serum intact FGF23 concentration was measured using an enzyme-linked immunosorbent assay (ELISA) kit (#EZMFGF23-43K, Millipore, Darmstadt, Germany) according to the manufacturer’s protocol. The level of serum vitamin D, phosphorus as well as renal tissue level of NGAL was measured by ELISA kit (ml038442-C, Mlbio, Shanghai, China; BS-E9480M1, Jinyou Technology, Beijing, China; CSB-E09410m, Cusabio, Wuhan, China; respectively), while serum creatinine was measured using a kit from Abcam (ab65340) according to the instructions of the manufacturers.

### Echocardiography

Both cardiac function and cardiac remodeling were evaluated by echocardiography using a Sequoia 512 system with a 17L-5 probe (Siemens, Germany). Mice were anesthetized by inhalation of 2% isoflurane and M-mode images were obtained with guidance by 2D parasternal short-axis images of the left ventricle at the level of the papillary muscles. LVEF, LVESd and LVEDd were measured from the M-mode images.

### Blood pressure measurement

The blood pressure was measured at 12 weeks after MI by pneumatic tail-cuff method (BP-2010 Blood Pressure Meter, Softron, Kyoto, Japan). Briefly, the instrument was preheated for 20 minutes to calibrate the pressure signal. Mice were put into the fixed frame and the tail was inserted into heating tube through pulse sensor. When the tail was rightly above the pulse sensor chip, the tail compression chip was adjusted to make the sensor chip close to the tail artery. The blood pressure was measured after the pulse was stable.

### Masson's trichrome

Paraffin-embedded sections of heart and kidney tissues were stained with Masson’s trichrome stain to evaluate fibrosis. In the heart, fibrotic lesions were measured with Image-Pro Plus 6.0 software as the Masson’s trichrome-positive area (blue staining) and the percent area was calculated, as described previously [42]. In the kidneys, the interstitial fibrosis score was graded as follows: 0) no interstitial fibrosis; 1) <25% fibrosis; 2) 25% to 50% fibrosis; and 3) >50% fibrosis [41]. Kidney sections were stained with hematoxylin and eosin stain by standard protocols. The extent of glomerular lesions was assessed semi-quantitatively, as reported elsewhere [41].

### Immunohistochemistry

Twelve weeks after starting the induction of CRS, heart and kidney tissues were harvested from each group, fixed in 4% paraformaldehyde, and embedded in paraffin. Then 4 μm sections were prepared for immunostaining. After antigen retrieval by heating in citrate buffer (pH 6.0), the sections were incubated with primary antibodies overnight at 4° C. FGF23 (1:50, Santa Cruz, CA, USA), Klotho (1:50, R&D Systems, MN, USA), FGFR4 and TGFβ (1:50) (Abcam, MA, USA). The sections were incubated with an HRP-labeled secondary antibody. The Dako EVision+System-HRP (DAB) was used to visualize the indicated protein staining. Images were captured using an upright microscope (Olympus, Japan).

### Isolation of RNA and PCR

Total RNA was extracted from cultured cells and from mouse heart and kidney tissues using a total RNA isolation system (R6934-01, Omega Bio-Tek, GA, USA). Reverse transcription was carried out in a reaction mixture (20 μL) containing 1μg of total RNA. PCR products were separated on agarose gels that were stained with ethidium bromide and digital images of the Klotho and GADPH bands were obtained. Gene expression levels of FGF23, FGFR4 and GAPDH in heart and kidney tissues were also determined using a Quantitect SYBR Green RT-PCR kit (RR420A, Takara Bio, Kusatsu/Shiga, Japan) and an Applied Light Cycler 480 system. Primer sequences are listed in Supplementary Table 2.

### Western blot analysis

Samples containing equal amounts of protein were separated by 10% sodium dodecyl sulfate-polyacrylamide gel electrophoresis and transferred to polyvinyl difluoride membranes. The membranes were blocked with 5% BSA at room temperature for 1 h, and then incubated overnight at 4° C with primary antibodies for the following targets: collagen I (1:1000, Bioss, Bejing, China), calcineurin A and FGFR4 (1:1000) (Abcam, MA, USA), vimentin, TGF-β, NFAT, p38 MAPK (1:1000) and phospho-p38 MAPK (1:500) (Cell Signaling Technology, MA, USA), p-GSK3α/β (1:500) and GSK3α/β (1:1000) (Santa Cruz, CA, USA), active-β-catenin (1:500) and β-catenin (1:1000) (Millipore, Darmstadt, Germany), Klotho and FGF23 (1:500) (R&D Systems, MN, USA), GAPDH (1:2000, Arigo, Shanghai, China). The reliability of the Klotho and FGF23 antibodies has been confirmed by previous study [6, 70–72]. Then the membranes were incubated with Dylight 800-labeled secondary antibodies and reaction products were detected with a western blotting detection system (Infrared Imaging System, LI-COR, USA). Relative expression was quantified by densitometry with Image J Analysis Software (National Institutes of Health, Bethesda, MD).

### Construction of recombinant AAV-FGF23 and injection into mice

A pAAV2/9-CMV-ZsGreen vector carrying FGF23 (NM022657, 756bp) and a negative control vector (NC) were generated by Vigene (Shandong, China). Then pAAV2/9-CMV-FGF23 or control virus (3.3×10^11^ viral genomes/mL) were administered to mice aged 6 weeks by direct injection into the left ventricular free wall at three sites (10 μL/site) using a syringe with a 30-gauge needle. MI surgery was performed 4 weeks later.

### Statistical analysis

Data are expressed as the mean ± SEM. Comparison of two groups was performed by Student’s t-test, while multiple comparisons were done by one-way or two-way ANOVA with Bonferroni’s multiple comparisons exact probability test. Significance was accepted at *P*<0.05.

## Supplementary Material

Supplementary Figures

Supplementary Tables
