# Supplementary material for: Excessive fibroblast growth factor 23 promotes renal fibrosis in mice with type 2 cardiorenal syndrome
Source: Aging (Albany NY). 2021 Jan 15;13(2):2982–3009. doi: 10.18632/aging.202448 (PMC7880350; doi:10.18632/aging.202448)
Supplement: Supplementary Figures [file aging-13-202448-s001.pdf]

SUPPLEMENTARY FIGURES

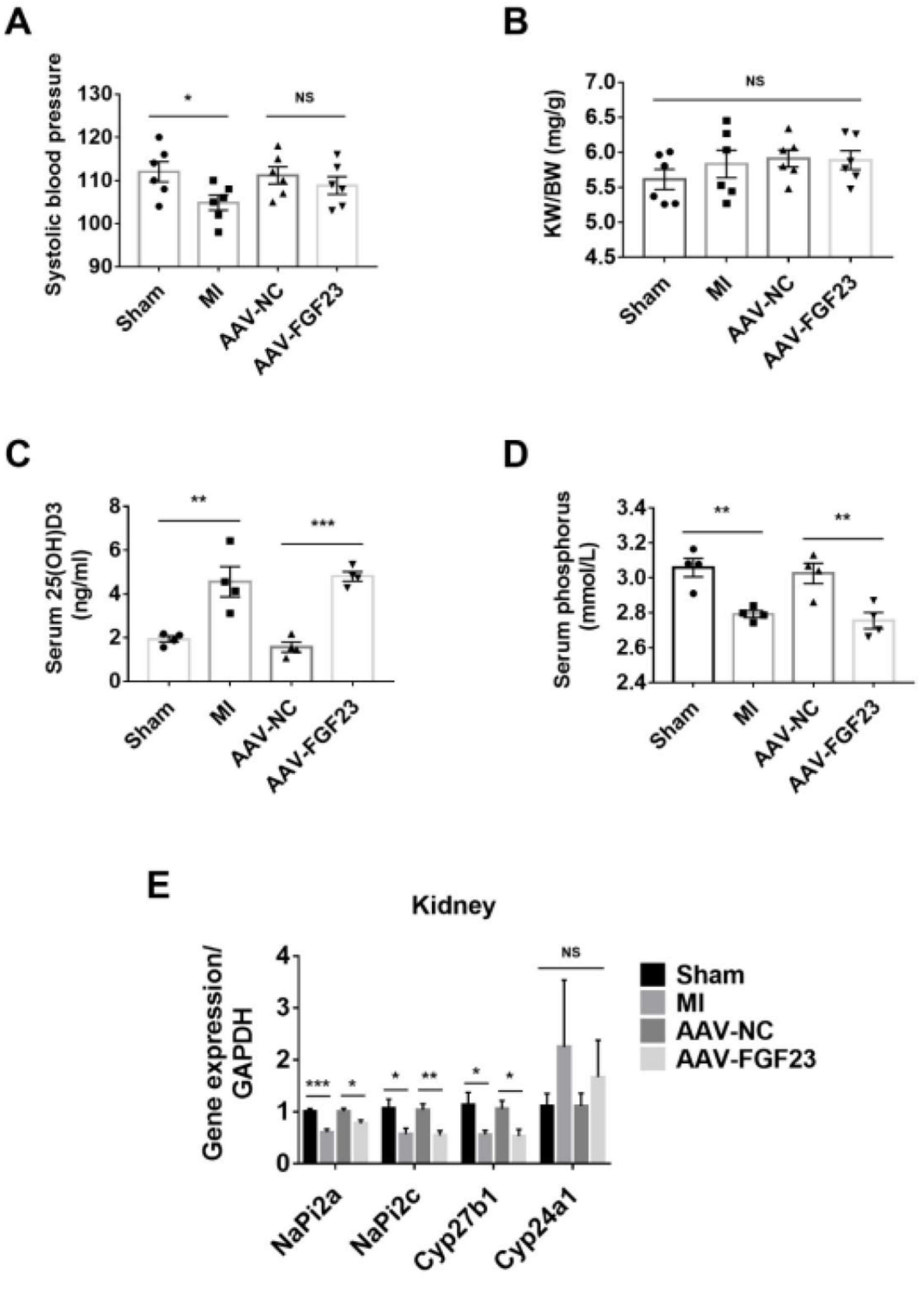

Supplementary Figure 1. The effects of MI or myocardial overexpression of FGF23 on hemodynamics, serological indicators and kidney related gene expression. (**A**) Systolic blood pressure. n = 6 per group. (**B**) Kidney weight/body weight ratio. n = 6 per group. Serum 25(OH)D3 (**C**) and serum phosphorus D (**D**) levels were measured by ELISA. n = 4 per group. (**E**) Quantitative real-time PCR for Napi2a, Napi2c, Cyp27b1 and Cyp24a1 mRNA in the kidneys. n = 6 per group, respectively. **P* < 0.05; ***P* < 0.01; ****P* < 0.001; NS, no statistical significance; Data are means ± SE.

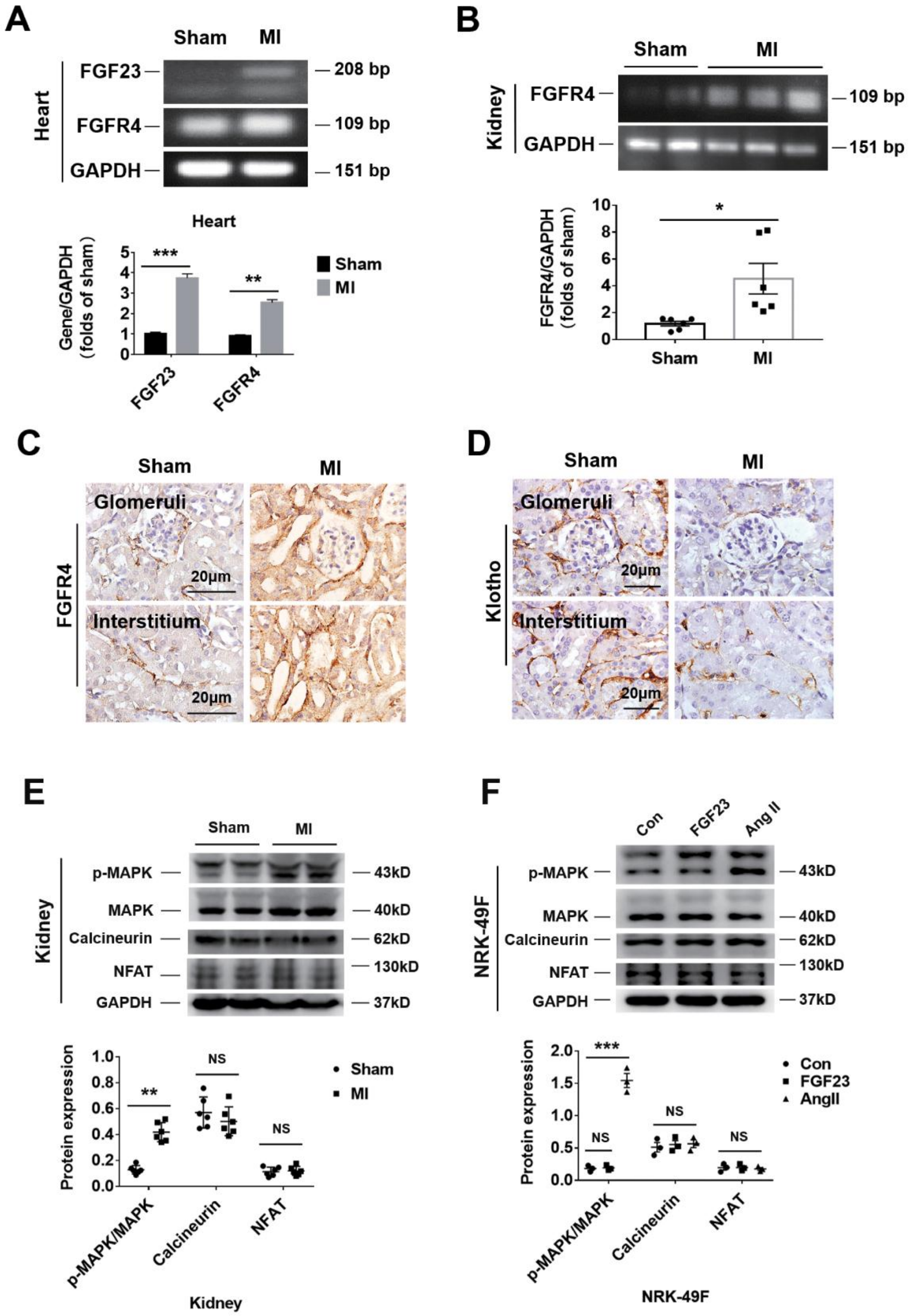

Supplementary Figure 2. FGF receptor 4 (FGFR4) was upregulated in the heart and kidneys and MAPK pathway was activated in the kidneys after induction of MI for 12 weeks. (**A**) Routine PCR and quantitative real-time PCR for FGF23 and FGFR4 mRNA in the heart. n = 6 per group. (**B**) Routine PCR and quantitative real-time PCR for FGFR4 mRNA in the kidneys. n = 6 per group, respectively. (**C**) Immunohistochemical staining displayed FGFR4 protein expression and localization in both the glomeruli and renal tubules. (**D**) Immunohistochemical staining showed reduced Klotho expression in the renal tubules of CRS mice compared with sham mice. (**E**) Western blot and semi-quantitative assessment of p-MAPK, MAPK, calcineurin and NFAT in the kidneys of CRS mice. n = 6 per group. (**F**) Western blot and semi-quantitative assessment of p-MAPK, MAPK, calcineurin and NFAT in cultured NRK-49F fibroblast cell line. n = 3 per group. **P* < 0.05; ***P* < 0.01; ****P* < 0.001; NS, no statistical significance; Data are means ± SE.

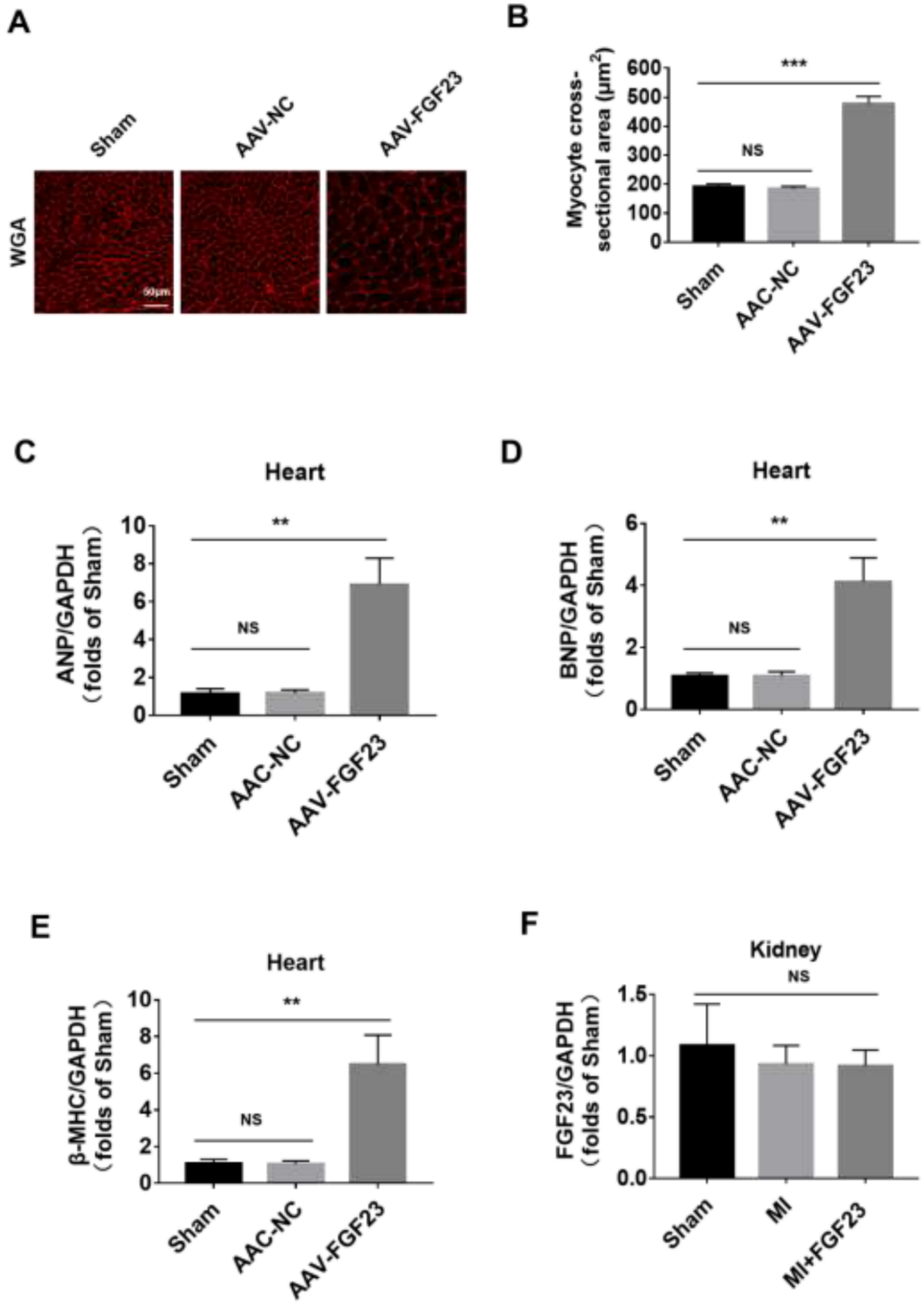

Supplementary Figure 3. Intramyocardial injection of FGF23 induced cardiac hypertrophy in mice. (**A**) The result of WGA-stained sections demonstrated that FGF23-induced cardiac hypertrophy. (**B**) Intramyocardial injection of FGF23 induced significantly increased cross-sectional surface area of individual cardiomyocytes. 20 cells from each animal were used to calculate its mean value of cross surface area of myocytes. Real-time PCR for ANP (**C**), BNP (**D**), β-MHC (**E**), and FGF23 (**F**). n = 20 in B, n = 6 in (**C–F**). $^{**}P < 0.01$; $^{***}P < 0.001$; NS, no statistical significance; Data are means ± SE.

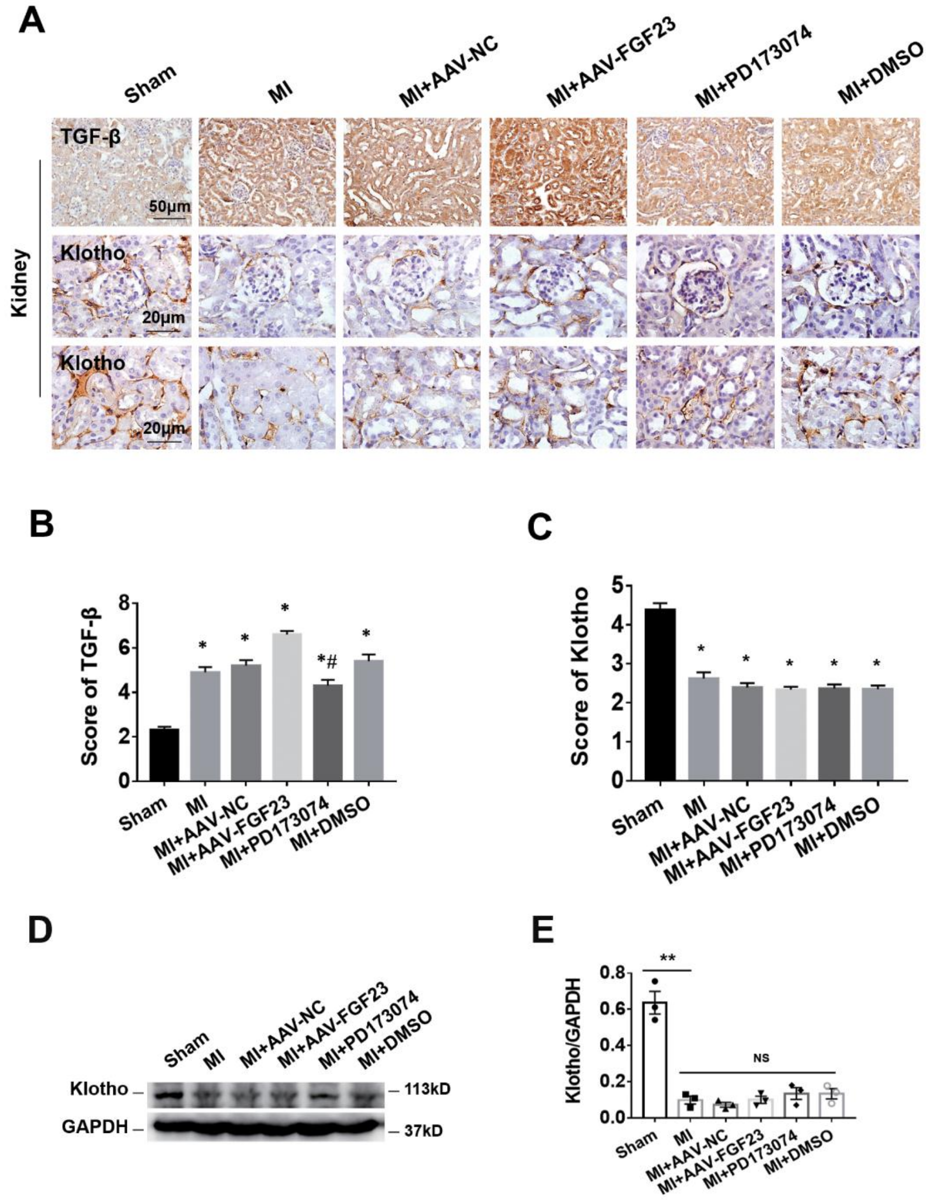

Supplementary Figure 4. Cardiac overexpression of FGF23 upregulated TGF-β and downregulated Klotho in the kidneys of mice with cardiorenal syndrome. Immunohistochemical staining was performed to detect renal expression of TGF-β and Klotho. (**A**) Representative images of TGF-β and Klotho immunostaining. (**B**) Semi-quantitative assessment of TGF-β. (**C**) Semi-quantitative assessment of Klotho. $^{\#}P$<0.05 vs. the MI+AAV-FGF23 group, n = 5 per group. (**D**) Western blot of Klotho. (**E**) Semi-quantitative assessment of Klotho. n = 3 per group. **P* < 0.05; ***P* < 0.01; NS, no statistical significance; Data are means ± SE.

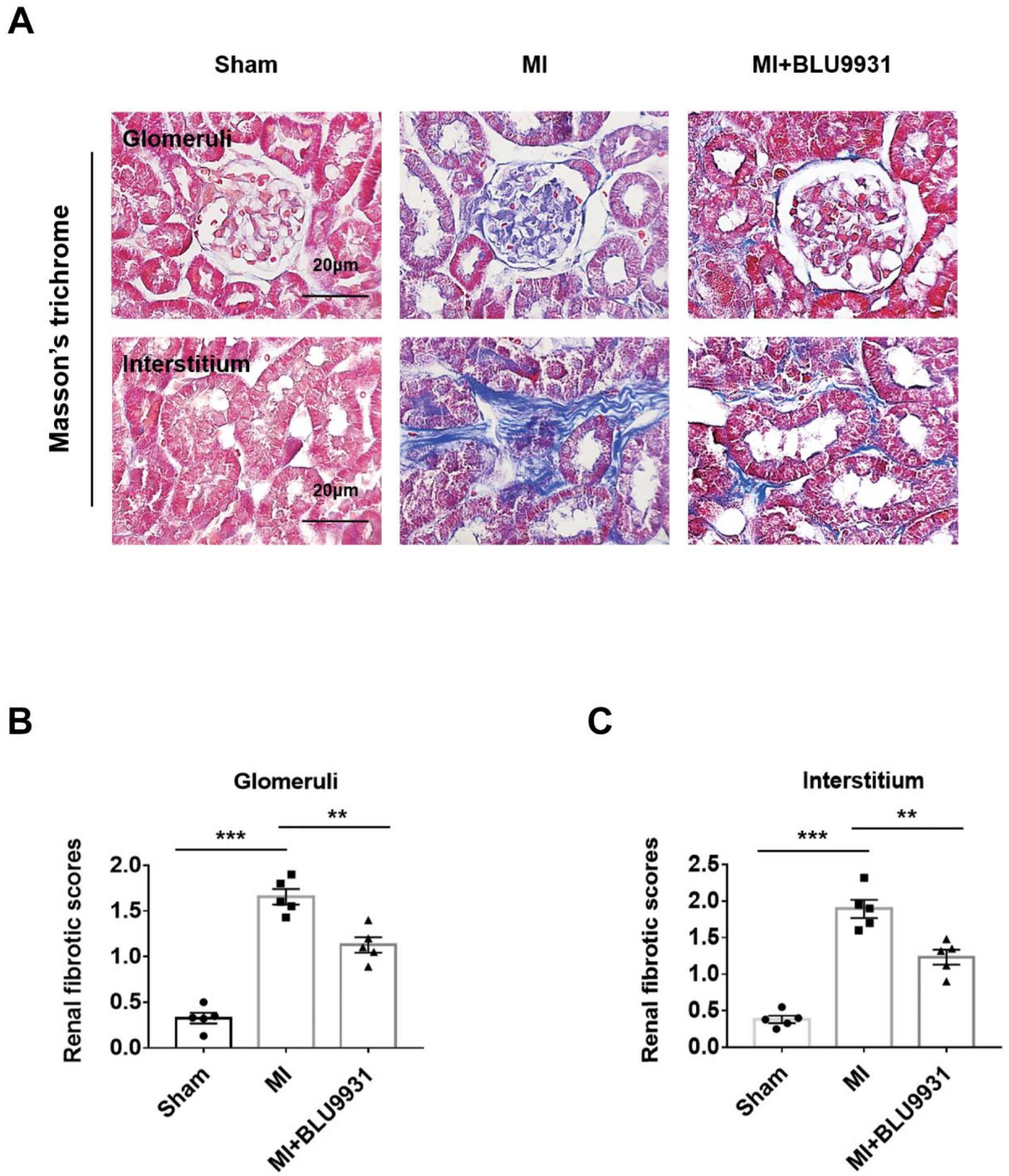

Supplementary Figure 5. FGFR4 antagonist BLU9931 also significantly inhibited MI-induced renal fibrosis. (**A**) Representative photomicrographs of renal fibrosis detected by Masson's trichrome stain in sham, MI and MI+BLU9931 groups. (**B**, **C**) Semi-quantitative assessment of glomerular and interstitial fibrosis. $^{**}P < 0.01$, $^{***}P < 0.001$. n = 5 per group. Data are means ± SE.

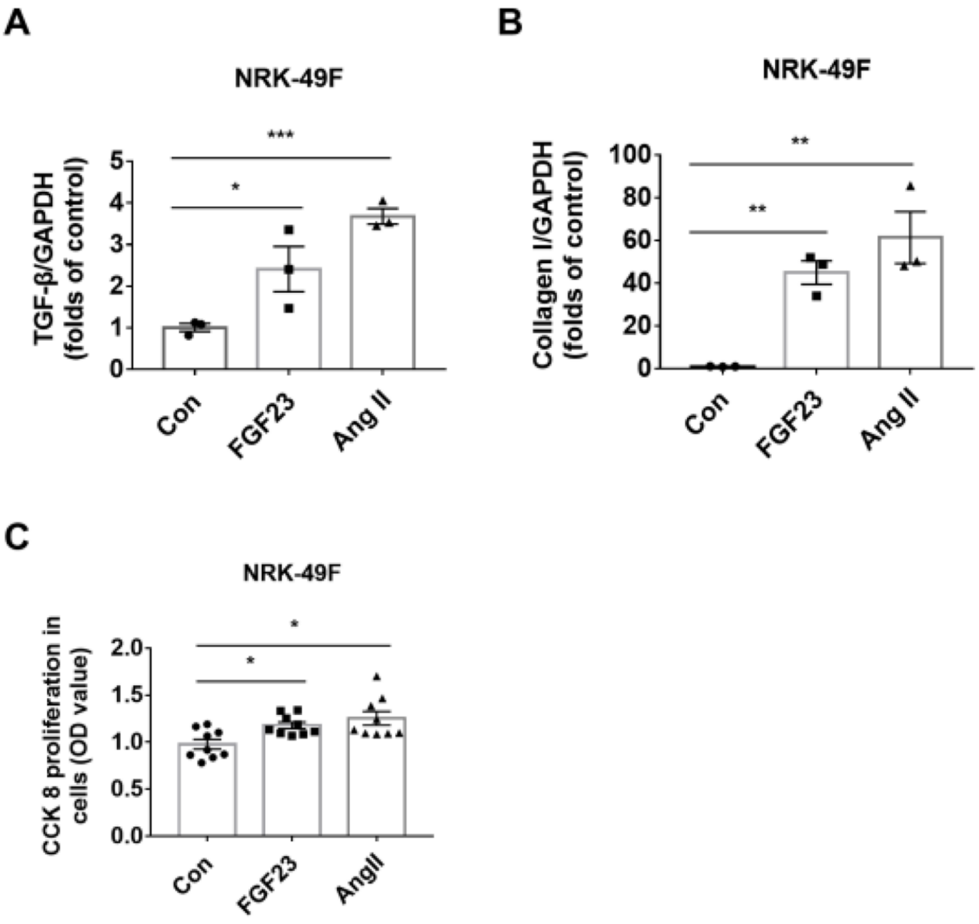

Supplementary Figure 6. Real-time PCR of TGF-β and Collagen I in cultured NRK-49F fibroblast cell line (**A**, **B**). n = 3 per group. (**C**) The result of CCK-8 showed that FGF23 promoted fibroblast proliferation., n = 9 per group. Con, control; FGF23, fibroblast growth factor 23 (100 ng/mL); Ang II, angitensin II (1 μM). **P* < 0.05; ***P* < 0.01; ****P* < 0.001; NS, no statistical significance; Data are means ± SE.
