# Supplementary material for: Excessive fibroblast growth factor 23 promotes renal fibrosis in mice with type 2 cardiorenal syndrome
Source: Aging (Albany NY). 2021 Jan 15;13(2):2982–3009. doi: 10.18632/aging.202448 (PMC7880350; doi:10.18632/aging.202448)
Supplement: Supplementary Tables [file aging-13-202448-s002.pdf]

SUPPLEMENTARY TABLES

Supplementary Table 1. Echocardiographic findings at 12 weeks after surgery.

Parameters	Sham(n=13)	MI (n=13)	*P* value
IVSd (mm)	0.66 ± 0.02	0.44 ± 0.02	0.000
LVEDd (mm)	2.85 ± 0.06	5.86 ± 0.31	0.000
LVPWd (mm)	0.66 ± 0.02	0.73 ± 0.03	0.099
LVESd (mm)	1.20 ± 0.06	5.10 ± 1.36	0.000
LVFS (%)	37.41 ± 1.24	14.53 ± 1.29	0.000
LVEF (%)	68.19 ± 1.65	28.78 ± 2.42	0.000

IVSd, interventricular septal thickness at diastole; LVEDd, left ventricular end-diastolic dimension; LVPWd, left ventricular diastolic posterior wall thickness; LVESd, left ventricular end-systolic dimension; LVFS, left ventricular fractional shortening; LVEF, left ventricular ejection fraction. Data are shown as the mean ± SE.

Supplementary Table 2. Sequences of primers for real-time-PCR.

Transcripts	Forward primer (5'–3')	Reverse primer (5'–3')	Size (bp)
FGF23 (mouse)	GCACTGCTAGAGCCTATCC	TGGCTCCTGTTATCACCAC	208
Collagen I (rat)	ACTCCAGTTGCTCCAGATGC	CCTGTCACCACCAGGCTACT	104
TGF-β (rat)	GGCGGTGCTCGCTTTGTA	GCGGGTGACTTCTTTGGC	118
FGFR4 (mouse)	GTGGCTGTGAAGATGCTGAA	ACCCAGCAGGTTGATGATGT	117
Klotho (rat)	CCTCATGGATGGTTTCGAGT	TTGGTAGAACAAGGCCGAAG	118
Klotho (mouse)	CAAACACACGGAACATGACC	CTGCAAGGCGATGGATATTT	125
GAPDH (mouse)	ATGTGTCCGTCGTGGATCTGA	TTGCTGTTGAAGTCGCAGGAG	108
GAPDH (rat)	TCCCATTCTTCCACCTTTGA	ATGTAGGCCATGAGGTCCAC	125
Napi2a (mouse)	CAATGCCATCCTATCCAACC	GACCATGCTGACAATGATGG	207
Napi2c (mouse)	ATCTCCGGTTCCATTCCAGG	GTAGAGGCTTCCCAGGAGTC	162
Cyp24a1 (mouse)	CCAAGGTCCGTGACATCCAA	GATGCACCGAGTCGAAGGAG	253
Cyp27b1 (mouse)	GAGGCAGTGAGTCGGTTCTC	ATTCTTCACCATCCGCCGTT	318
